# Impact of Risk Factors for Specific Causes of Death in the First and Subsequent Years of Antiretroviral Therapy Among HIV-Infected Patients

**DOI:** 10.1093/cid/ciu261

**Published:** 2014-04-24

**Authors:** Suzanne M. Ingle, Margaret T. May, M. John Gill, Michael J. Mugavero, Charlotte Lewden, Sophie Abgrall, Gerd Fätkenheuer, Peter Reiss, Michael S. Saag, Christian Manzardo, Sophie Grabar, Mathias Bruyand, David Moore, Amanda Mocroft, Timothy R. Sterling, Antonella D'Arminio Monforte, Victoria Hernando, Ramon Teira, Jodie Guest, Matthias Cavassini, Heidi M. Crane, Jonathan A. C. Sterne

**Affiliations:** 1 Schoolof Social and Community Medicine, University of Bristol, United Kingdom; 2 Division of Infectious Diseases, University of Calgary, Canada; 3 Division of Infectious Disease, Department of Medicine, University of Alabama, Birmingham; 4 INSERM, Centre INSERM U897-Epidemiologie-Biostatistique, Bordeaux; 5 Université Bordeaux, Institut de Santé Publique, d'Epidémiologie et de Developpement (ISPED); 6 UPMC Université Paris 06, UMR_S 943; 7 INSERM, UMR_S 943, Paris; 8 Service des maladies infectieuses et tropicales, AP-HP, Hôpital Avicenne, Bobigny, France; 9 Department of Internal Medicine, University of Cologne, Germany; 10 Stichting HIV Monitoring, and Division of Infectious Diseases and Department of Global Health, Amsterdam Institute for Global Health and Development, Academic Medical Center, University of Amsterdam, The Netherlands; 11 Infectious Diseases Service, Hospital Clinic–IDIBAPS, Barcelona, Spain; 12 AP-HP, Hôpital Cochin, Unité de Biostatistique et Epidémiologie, Paris; 13 Université Paris Descartes; 14 INSERM, ISPED, Centre Inserm U897-Epidemiologie-Biostatistique, Bordeaux, France; 15 BC Centre for Excellence in HIV/AIDS, Department of Medicine, University of British Columbia, Vancouver, Canada; 16 Research Department of Infection and Population Health, University College London, United Kingdom; 17 Vanderbilt University School of Medicine, Nashville, Tennessee; 18 Clinic of Infectious Diseases and Tropical Medicine, San Paolo Hospital, University of Milan, Italy; 19 Red de Investigación en Sida, Centro Nacional de Epidemiología, Instituto de Salud Carlos III, Madrid; 20 CIBER de Epidemiología y Salud Pública, Madrid; 21 Unit of Infectious Diseases, Hospital Sierrallana, Torrelavega, Spain; 22 HIV Atlanta VA Cohort Study, Atlanta; 23 Veterans Affairs Medical Center, Decatur, Georgia; 24 Service of Infectious Diseases, Lausanne University Hospital and University of Lausanne, Switzerland; 25 Clinical Epidemiology and Health Services Research Core, Center for AIDS Research, University of Washington, Seattle

**Keywords:** HIV, cause-specific mortality, antiretroviral therapy

## Abstract

***Background.*** Patterns of cause-specific mortality in individuals infected with human immunodeficiency virus type 1 (HIV-1) are changing dramatically in the era of antiretroviral therapy (ART).

***Methods.*** Sixteen cohorts from Europe and North America contributed data on adult patients followed from the start of ART. Procedures for coding causes of death were standardized. Estimated hazard ratios (HRs) were adjusted for transmission risk group, sex, age, year of ART initiation, baseline CD4 count, viral load, and AIDS status, before and after the first year of ART.

***Results.*** A total of 4237 of 65 121 (6.5%) patients died (median, 4.5 years follow-up). Rates of AIDS death decreased substantially with time since starting ART, but mortality from non-AIDS malignancy increased (rate ratio, 1.04 per year; 95% confidence interval [CI], 1.0–1.1). Higher mortality in men than women during the first year of ART was mostly due to non-AIDS malignancy and liver-related deaths. Associations with age were strongest for cardiovascular disease, heart/vascular, and malignancy deaths. Patients with presumed transmission through injection drug use had higher rates of all causes of death, particularly for liver-related causes (HRs compared with men who have sex with men: 18.1 [95% CI, 6.2–52.7] during the first year of ART and 9.1 [95% CI, 5.8–14.2] thereafter). There was a persistent role of CD4 count at baseline and at 12 months in predicting AIDS, non-AIDS infection, and non-AIDS malignancy deaths. Lack of viral suppression on ART was associated with AIDS, non-AIDS infection, and other causes of death.

***Conclusions.*** Better understanding of patterns of and risk factors for cause-specific mortality in the ART era can aid in development of appropriate care for HIV-infected individuals and inform guidelines for risk factor management.

## Abstract (precis)

Among HIV-infected patients who initiated antiretroviral therapy (ART), patterns of cause-specific death varied by ART duration and were strongly related to age, sex, and transmission risk group. Deaths from non-AIDS malignancies were much more frequent than those from cardiovascular disease.

Antiretroviral therapy (ART) has dramatically reduced mortality for people living with human immunodeficiency virus (HIV), or PLWH, allowing life expectancy for those successfully treated to approach that of the general population [1–3]. However, overall mortality among PLWH remains higher than in the general population [4–6]. As the HIV-infected population ages and duration on ART increases, the distribution of causes of death and associations with patient characteristics are changing. The proportion of AIDS-related deaths in PLWH has decreased in the ART era [7, 8], and decreases with duration on ART [9]. In high-income countries, there is concern over excess mortality from cancer [6, 10–12] and cardiovascular disease (CVD) [6]. Appropriate management of HIV disease and associated comorbidities may result in further decreases in mortality, but will depend on a detailed understanding of causes of death.

Based on a large collaboration of cohorts of PLWH in whom causes of death were classified according to standardized procedures, we assessed how patterns of causes of death change with time since starting ART. We estimated associations of patient characteristics with specific causes of death in the first year after starting ART and in subsequent years.

## METHODS

### Cohorts and Patients

The Antiretroviral Therapy Cohort Collaboration (ART-CC) is a collaboration of cohort studies of PLWH from Europe and North America [13] (available at: http://www.bris.ac.uk/art-cc). Prospective cohorts were eligible if they had enrolled at least 100 HIV type 1 (HIV-1)–infected patients aged ≥16 years who were not previously exposed to antiretroviral drugs and started ART with at least 3 drugs, including nucleoside reverse transcriptase inhibitors, protease inhibitors, or nonnucleoside reverse transcriptase inhibitors, with a median follow-up of ≥1 year. All cohorts use quality control procedures. The database was updated in September 2010. Institutional review boards approved data collection at all sites according to local requirements. The National Health Service Health Research Authority South West–Cornwall and Plymouth Research Ethics Committee, United Kingdom, has approved the ART-CC study (REC reference 12/SW/0253).

### Coding Causes of Death

Information on cause of death was recorded by 16 cohorts (Appendix) either as *International Classification of Diseases, Ninth Revision* (*ICD-9*) or *Tenth Revision* (*ICD-10*), Classification based on the Coding of Death in HIV (CoDe) project (available at: http://www.chip.dk/CoDe/tabid/55/Default.aspx), or free text. We adapted the CoDe protocol [14] to classify causes of death into mutually exclusive categories. Clinicians classified deaths using summary tables of patient details that included *ICD-9/ICD-10* codes or free text for cause of death, patient characteristics at ART initiation (age, sex, transmission risk group, AIDS-defining conditions, and hepatitis C status), time from ART initiation to death, AIDS-defining conditions after starting ART, latest CD4 (within 6 months of death), and whether a patient was on ART at time of death. A computer algorithm developed by the Mortalité 2000–2005 Study Group [15] classified deaths using *ICD-10* codes, when available. When *ICD-10* codes were not available, 2 clinicians independently classified each death. Disagreements between clinicians and/or computer-assigned codes were resolved via panel discussion as per the CoDe protocol described previously [9]. Further information on rules to classify deaths is provided in the Supplementary Data.

We grouped causes of death with a frequency of <20 as “other.” AIDS was defined according to the 1993 Centers for Disease Control and Prevention classification [16] and included CoDe 01 (unspecified AIDS), 01.1 (AIDS infection), and 01.2 (AIDS malignancy). We grouped deaths as AIDS, non-AIDS infection, liver-related (hepatitis and liver failure, CoDe 03 and 14), non-AIDS nonhepatitis malignancy (CoDe 04), cardiovascular disease (myocardial infarction/ischemic heart disease, CoDe 08, and stroke, CoDe 09), heart/vascular disease (heart failure/unspecified, and other heart disease, CoDe 24), respiratory disease (chronic obstructive lung disease, CoDe 13 and 25), renal failure (CoDe 15), disease of the central nervous system (CoDe 23), unnatural deaths (accident/violent, suicide, and overdose, CoDe 16, 17, and 19), and other (CoDe 90).

### Statistical Analysis

Follow-up time was calculated from date of starting ART (“baseline”) to the earliest of death, last clinic follow-up plus 3 months (because cohorts scheduled visits every 6 months and therefore using the last clinic follow-up date would underestimate follow-up time), or cohort-specific administrative censoring date (“database close”). Crude mortality rates for each cause of death were compared for all patients and by time since starting ART (<0.5, 0.5–0.99, 1–1.99, 2–4.99, and 5–10 years). Crude hazard ratios (HRs) were derived according to age, baseline CD4 count, and risk group. Median CD4 count at death (interquartile range [IQR]) was calculated for each cause of death. To investigate changes in patterns of cause of death over time, and to avoid violation of the proportional hazards assumption, separate Cox models were used to estimate HRs in the first year of ART and in subsequent years. Models were adjusted for sex, risk group (men who have sex with men [MSM], injection drug users [IDUs], heterosexuals, and other [includes transmission through blood or other unknown routes]), current age (per decade), baseline CD4 count (<50, 50–99, 100–199, 200–349, ≥350 cells/µL), baseline viral load (<100 000 copies, ≥100 000 copies), AIDS prior to ART, and year of ART initiation (1996–1999, 2000–2003, 2004–2009), stratified by cohort. HRs after the first year were adjusted for 12-month CD4 count and viral load (closest measure to 12 months after ART start, within ±3 months) and for AIDS status at 12 months. Patients were classified as having AIDS at 12 months if they had an AIDS diagnosis any time up to 12 months after ART start. Analyses were repeated accounting for competing risks using the method of Fine and Gray [17].

## RESULTS

Of 65 121 patients followed up between 1996 and 2009, 4237 (6.5%) died during 327 535 person-years of follow-up (median, 4.5 years [IQR, 2.0–7.8]), giving a mortality rate of 12.9 deaths per 1000 person-years (95% confidence interval [CI], 12.6–13.3). A total of 1225 (28.9%) deaths occurred in the first year of ART mortality rate 20.0 per 1000 person-years (95% CI, 18.9–21.2) and 3012 (71.1%) after the first year mortality rate 11.3 per 1000 person-years (95% CI, 10.9–11.7). Specific cause of death was classifiable for 3574 (84.4%) deaths (1057 in the first year and 2517 after). Compared with patients whose deaths were classified, those with unclassifiable deaths were older, had higher baseline CD4 and lower viral load. A total of 1496 (41.9%) of the classified deaths were due to AIDS (AIDS infection: 557, nonspecific AIDS: 550, AIDS malignancy: 389), 461 (12.9%) to non-AIDS malignancy, and 349 (9.8%) to unnatural causes. Patients were lost to follow-up if they had no visit recorded in the year prior to database close. Of 65 121 patients, 23 794 were lost to follow-up (4813 in the first year since ART initiation and 18 981 in subsequent years), giving a rate of 7.26 per 100 person-years (95% CI, 7.17–7.36), ranging from 2.02 to 10.39 across cohorts. Compared with patients remaining in care, those lost to follow-up were younger, more likely to be IDUs, initiated in earlier years, and had higher baseline CD4 and lower viral load.

Table 1 shows patient characteristics at baseline, overall and according to whether death was AIDS or non-AIDS related. The majority of patients were male and infected through sexual contact. Median age was 37 years (IQR, 31–44 years), the median CD4 count was 217 cells/µL (IQR, 96–340), and the median viral load was 70 000 copies/mL (IQR, 14 000–213 684). A total of 14 202 patients (21.8%) had an AIDS diagnosis before ART. Compared with patients who died of AIDS, those who died from non-AIDS causes were older, were more likely to be IDUs, had higher baseline CD4 count and lower viral load, and were less likely to have AIDS prior to ART.

**Table 1.** Patient Characteristics at Antiretroviral Therapy Start for the Whole Cohort, and According to Cause of Death

Characteristic		Non-AIDS Deaths	AIDS Deaths
	Whole Cohort N = 65 121	n = 2078 (58.1%)	n = 1496 (41.9%)
Sex			
Male	46 798 (71.9)	1702 (81.9)	1175 (78.5)
Female	18 323 (28.1)	376 (18.1)	321 (21.5)
Age, y			
16–29	12 322 (18.9)	158 (7.6)	190 (12.7)
30–39	27 682 (42.5)	754 (36.3)	600 (40.1)
40–49	16 407 (25.2)	628 (30.2)	401 (26.8)
50–59	6339 (9.7)	348 (16.8)	200 (13.4)
≥60	2371 (3.6)	190 (9.1)	105 (7.0)
Transmission risk group			
MSM	21 177 (32.5)	482 (23.2)	392 (26.2)
IDU	10 396 (16.0)	724 (34.8)	348 (23.3)
Heterosexual	26 668 (41.0)	592 (28.5)	458 (30.6)
Other	6880 (10.6)	280 (13.5)	298 (19.9)
CD4 at ART start, cells/µL			
<50	10 210 (15.7)	432 (20.8)	602 (40.2)
50–99	6462 (9.9)	274 (13.2)	256 (17.1)
100–199	13 106 (20.1)	459 (22.1)	299 (20.0)
200–349	19 971 (30.7)	513 (24.7)	231 (15.4)
≥350	15 372 (23.6)	400 (19.3)	108 (7.2)
Viral load at ART start, copies/mL			
<10 000	14 157 (21.7)	341 (16.4)	194 (13.0)
10 000–99 999	23 600 (36.2)	664 (32.0)	401 (26.8)
≥100 000	27 364 (42.0)	1073 (51.6)	901 (60.2)
AIDS diagnosis at ART start			
No AIDS	50 919 (78.2)	1491 (71.7)	705 (47.1)
AIDS	14 202 (21.8)	587 (28.3)	791 (52.9)
Period of ART initiation			
1996–1999	19 720 (30.3)	1065 (51.3)	687 (45.9)
2000–2003	22 528 (34.6)	691 (33.3)	535 (35.8)
2004–2009	22 873 (35.1)	322 (15.5)	274 (18.3)

Data are presented as No. (%).

Abbreviations: ART, antiretroviral therapy; IDU, injection drug user; MSM, men who have sex with men.

Table 2 shows rates of causes of death for all patients, and numbers of deaths and crude HRs according to baseline CD4 (<200 vs ≥200 cells/µL), age (<60 vs ≥60 years), and transmission risk group. The rate of AIDS death was 4.6 (95% CI, 4.3–4.8) per 1000 person-years. Patients with baseline CD4 count ≥200 cells/µL experienced lower rates of each cause of death than those with baseline CD4 count <200 cells/µL, except for unnatural causes and suicide. Rates of cause-specific mortality were higher among older than younger patients, except for unnatural and liver-related deaths. The higher rate of liver-related deaths in younger patients may be explained by the higher proportion of IDUs in this group. Older people had higher rates of malignancy and CVD. Rates for MSM and heterosexuals were broadly similar, although MSM had a higher rate of AIDS malignancy. Patients with presumed transmission via injection drug use had higher rates of all causes of death than other risk groups, particularly deaths from infection, liver disease, and substance abuse.

**Table 2.** Crude Cause-Specific Mortality Rates per 1000 Person-years and Numbers of Deaths, Stratified by Baseline CD4, Age, and Transmission Risk Group

	All Patients		Baseline CD4, Cells/µL			Age at Baseline, y			Transmission Risk Group				
			<200^a^	≥200		<60^a^	≥60		MSM^a^	IDU		Heterosexual	
Cause of Death	No.	Rate	No.	No.	HR	No.	No.	HR	No.	No.	HR	No.	HR
All causes	4237	12.9 (12.6–13.3)	2722	1515	0.5 (.4–.5)	3873	364	2.9 (2.6–3.2)	1037	1247	2.4 (2.2–2.6)	1247	1.0 (.9–1.1)
AIDS	1496	4.6 (4.3–4.8)	1157	339	0.2 (.2–.3)	1391	105	2.2 (1.8–2.7)	392	348	1.8 (1.6–2.1)	458	1.0 (.8–1.1)
AIDS (nonspecific)	550	1.7 (1.5–1.8)	428	122	0.2 (.2–.3)	501	49	3.0 (2.2–4.0)	119	176	3.0 (2.4–3.8)	169	1.2 (.9–1.5)
AIDS infection	557	1.7 (1.6–1.8)	457	100	0.2 (.1–.2)	530	27	1.5 (1.0–2.2)	113	126	2.3 (1.7–2.9)	193	1.4 (1.1–1.8)
AIDS malignancy	389	1.2 (1.1–1.3)	272	117	0.4 (.3–.4)	360	29	2.3 (1.6–3.4)	160	46	0.6 (.4–.8)	96	0.5 (.4–.6)
Non-AIDS	2078	6.3 (6.1–6.6)	1165	913	0.6 (.6–.7)	1888	190	3.2 (2.7–3.7)	482	724	3.0 (2.7–3.4)	592	1.1 (.9–1.2)
Malignancy (non-AIDS nonhepatitis)	461	1.4 (1.3–1.5)	266	195	0.6 (.5–.7)	406	55	4.4 (3.3–5.8)	147	91	1.2 (1.0–1.6)	174	1.0 (.8–1.3)
Infection	314	1.0 (.9–1.1)	208	106	0.4 (.3–.5)	286	28	3.0 (2.1–4.5)	51	109	4.3 (3.1–6.0)	99	1.7 (1.2–2.3)
Liver related (all, includes liver cancer)	302	0.9 (.8–1.0)	172	130	0.6 (.5–.8)	296	6	0.6 (.3–1.4)	35	184	10.6 (7.4–15.2)	51	1.2 (.8–1.9)
Hepatitis	222	0.7 (.6–.8)	129	93	0.6 (.4–.8)	219	3	0.4 (.1–1.4)	19	161	17.0 (10.6–27.4)	21	1.0 (.5–1.8)
Liver failure	80	0.2 (.2–.3)	43	37	0.7 (.5–1.1)	77	3	1.2 (.4–3.8)	16	23	2.9 (1.5–5.5)	30	1.6 (.9–2.9)
CVD	160	0.5 (.4–.6)	83	77	0.7 (.5–1.0)	127	33	8.3 (5.7–12.2)	53	27	1.0 (.6–1.6)	49	0.8 (.5–1.2)
MI/IHD	106	0.3 (.3–.4)	55	51	0.7 (.5–1.1)	86	20	7.4 (4.6–12.1)	40	21	1.1 (.6–1.8)	25	0.5 (.3–.9)
Stroke	54	0.2 (.1–.2)	28	26	0.7 (.4–1.3)	41	13	10.2 (5.4–19.0)	13	6	0.9 (.4–2.4)	24	1.6 (.8–3.1)
Heart/vascular	131	0.4 (.3–.5)	75	56	0.6 (.4–.8)	115	16	4.3 (2.6–7.3)	28	43	3.1 (1.9–5.0)	41	1.3 (.8–2.0)
Unnatural (all)	349	1.1 (1.0–1.2)	147	202	1.1 (.9–1.4)	339	10	0.9 (.5–1.7)	80	164	4.1 (3.2–5.4)	57	0.6 (.4–.9)
Suicide	97	0.3 (.2–.4)	32	65	1.6 (1.1–2.5)	95	2	0.7 (.2–2.7)	43	27	1.3 (.8–2.0)	20	0.4 (.2–.7)
Substance abuse	191	0.6 (.5–.7)	85	106	1.0 (.8–1.3)	186	5	0.8 (.3–2.0)	18	117	13.1 (8.0–21.5)	27	1.3 (.7–2.3)
Other violent	61	0.2 (.1–.2)	30	31	0.8 (.5–1.4)	58	3	1.6 (.5–5.2)	19	20	2.1 (1.1–4.0)	10	0.5 (.2–1.0)
Respiratory	61	0.2 (.1–.2)	33	28	0.7 (.4–1.1)	49	12	7.9 (4.2–14.9)	15	19	2.5 (1.3–5.0)	20	1.2 (.6–2.3)
Renal	37	0.1 (.1–.2)	30	7	0.2 (.1–.4)	31	6	6.0 (2.5–14.5)	5	4	1.6 (.4–6.0)	17	2.9 (1.1–7.9)
CNS	41	0.1 (.1–.2)	30	11	0.3 (.1–.6)	37	4	3.3 (1.2–9.3)	14	11	1.6 (.7–3.5)	8	0.5 (.2–1.2)
Other^b^	222	0.7 (.6–.8)	121	101	0.7 (.5–.9)	202	20	3.1 (2.0–5.0)	54	72	2.7 (1.9–3.8)	76	1.2 (.9–1.7)
Unknown	663	2.0 (1.9–2.2)	400	263	0.5 (.5–.6)	594	69	3.6 (2.8–4.6)	163	175	2.2 (1.7–2.7)	197	1.0 (.8–1.3)

Ranges in parentheses are 95% confidence intervals.

Abbreviations: CNS, central nervous system; CVD, cardiovascular disease; HR, hazard ratio; IDU, injection drug user; IHD, ischaemic heart disease; MI, myocardial infarction; MSM, men who have sex with men.

^a^ Baseline category for comparison.

^b^ “Other” deaths were diabetes (5), pancreatitis (6), lactic acidosis (11), gastrointestinal hemorrhage (18), pulmonary hypertension (4), lung embolus (18), euthanasia (1), hematological (14), psychiatric (8), digestive (16), skin/motor system disease (1), urogenital (1), obstetric (1), congenital (1), other (117).

**Figure 1.**
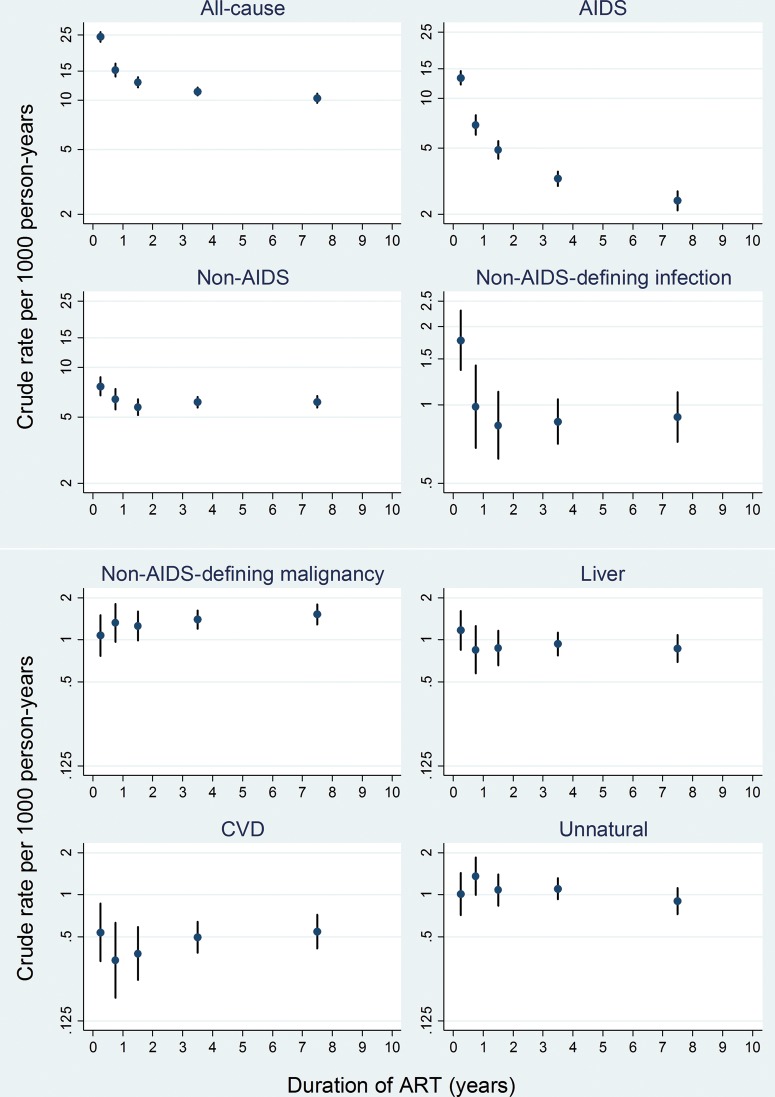
Cause-specific crude mortality rate per 1000 person-years by duration of antiretroviral therapy. Vertical axis is on log scale. Abbreviations: ART, antiretroviral therapy; CVD, cardiovascular disease.

Figure 1 shows rates of selected causes of death by time since starting ART. All-cause mortality rates decreased from 24.3 (95% CI, 22.7–26.1) per 1000 person-years in the first 6 months of ART to 10.2 (95% CI, 9.6–10.9) after 5 years. This was largely due to substantial decreases in AIDS-related death, from 13.2 (95% CI, 12.0–14.5) per 1000 person-years in the first 6 months of ART to 2.4 (95% CI, 2.1–2.8) after 5 years. Rates of non-AIDS infection also decreased with time since starting ART, from 1.8 (95% CI, 1.4–2.3) in the first 6 months to 0.9 (95% CI, 0.7–1.1) after 5 years. By contrast, mortality from non-AIDS malignancy increased slightly, from 1.1 (95% CI, 0.8–1.5) per 1000 person-years in the first 6 months to 1.5 (95% CI, 1.3–1.8) after 5 years: the adjusted (for sex, year of ART start, age, CD4 and viral load, and stratified on cohort) rate ratio was 1.04 (95% CI, 1.0–1.1) per year on ART. Rates of CVD deaths had a U-shaped relationship with ART duration, with the lowest rates observed between 1 and 2 years after starting ART.

**Figure 2.**
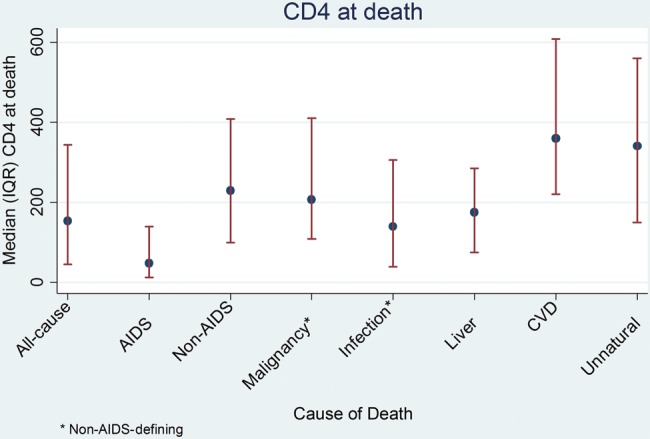
Latest CD4 count before death within 6 months of death. Abbreviations: CVD, cardiovascular disease; IQR, interquartile range.

CD4 measures within 6 months of death were available for 3215 patients. Median CD4 at death (median time from CD4 to death, 52 days [IQR, 24–94 days]) was 154 cells/µL (IQR, 45–344). CD4 at death was lowest for those who died of AIDS-related causes (median, 48 [IQR, 13–140], due partly to the use of CD4 in assigning causes of death), and highest for those who died of CVD (median, 360 [IQR, 221–608]) and unnatural causes (median, 340 [IQR, 150–560]) (Figure 2).

### Associations of Risk Factors With Mortality According to Time Since Starting ART

Table 3 shows associations of risk factors with cause-specific mortality in the first year of ART. Of 1225 deaths, 168 could not be classified and 82 were from other causes. Compared with MSM, IDUs had higher rates of each cause of death: rates of liver-related (HR, 18.1 [95% CI, 6.2–52.7]), substance abuse (HR, 16.3 [95% CI, 3.7–72.3]), and heart/vascular (HR, 6.8 [95% CI, 2.2–21.0]) death were particularly elevated. Lower mortality in women than men was mostly due to lower rates of non-AIDS malignancy (HR, 0.3 [95% CI, 0.1–0.7]) and liver-related (HR, 0.5 [95% CI, 0.2–1.0]) death. There was little evidence that baseline viral load was associated with any specific cause of death in the first year. AIDS before starting ART was strongly associated with rates of AIDS (HR, 4.3 [95% CI, 3.6–5.2]), non-AIDS infection (HR, 2.8 [95% CI, 1.7–4.6]), and liver-related (HR, 1.9 [95% CI, 1.1–3.4]) death. Rates of death from substance abuse were lower after 1999.

**Table 3.** Adjusted^a^ Hazard Ratios From Cox Regression Models in the First Year of Antiretroviral Therapy (N = 65121)

Risk Factor	No.	AIDS	Non-AIDS Malignancy	Non-AIDS Infection	Liver Related	CVD	Heart/Vascular	Suicide	Substance Abuse	Accident/ Violent^b^
No. (%) of deaths		625 (51)	73 (6)	85 (7)	62 (5)	27 (2)	31 (3)	21 (2)	41 (3)	10 (1)
Transmission risk group										
MSM	21 177	1	1	1	1	1	1	1	1	
IDU	10 396	1.13 (.86–1.49)	1.46 (.66–3.21)	2.47 (1.21–5.04)	18.05 (6.18–52.72)	0.52 (.11–2.52)	6.81 (2.21–20.97)	1.32 (.33–5.31)	16.31 (3.68–72.31)	
Heterosexual	26 668	0.90 (.72–1.12)	1.02 (.57–1.84)	1.26 (.66–2.38)	4.25 (1.40–12.95)	0.41 (.14–1.18)	2.53 (.86–7.42)	0.83 (.26–2.61)	2.93 (.56–15.33)	
Other	6880	1.91 (1.51–2.41)	1.34 (.65–2.77)	2.66 (1.33–5.31)	6.50 (1.93–21.83)	0.62 (.19–2.00)	1.37 (.26–7.31)	1.24 (.30–5.13)	6.32 (1.18–33.68)	
Sex										
Male	46 798	1	1	1	1	1	1	1	1	
Female	18 323	0.91 (.73–1.13)	0.29 (.12–.70)	0.82 (.46–1.45)	0.50 (.24–1.01)	1.62 (.56–4.74)	0.46 (.17–1.26)	0.17 (.02–1.39)	1.40 (.70–2.83)	
Current age (Per decade)	65 121	1.30 (1.21–1.40)	2.13 (1.76–2.59)	1.58 (1.30–1.91)	1.49 (1.16–1.92)	2.62 (1.94–3.54)	1.71 (1.24–2.36)	1.30 (.86–1.97)	1.24 (.87–1.76)	
CD4 count at ART initiation, cells/µL										
<50	10 210	1	1	1	1	1	1	1	1	
50–99	6462	0.72 (.58–.89)	0.72 (.33–1.57)	0.58 (.29–1.15)	1.31 (.55–3.14)	0.89 (.21–3.76)	1.08 (.30–3.89)	0.18 (.02–1.50)	0.73 (.13–4.01)	
100–199	13 106	0.49 (.39–.61)	0.54 (.27–1.10)	0.76 (.43–1.35)	1.33 (.61–2.93)	1.44 (.45–4.58)	1.67 (.58–4.82)	0.08 (.01–.69)	2.06 (.63–6.71)	
200–349	19 971	0.28 (.21–.37)	0.51 (.25–1.02)	0.46 (.23–.92)	0.97 (.42–2.24)	1.04 (.30–3.57)	0.71 (.20–2.45)	0.34 (.10–1.16)	1.33 (.38–4.60)	
≥350	15 372	0.09 (.05–.15)	0.25 (.10–.65)	0.22 (.07–.65)	0.68 (.25–1.83)	0.38 (.07–2.17)	0.64 (.16–2.58)	0.55 (.16–1.92)	2.13 (.63–7.27)	
Viral load at ART initiation, log copies/mL										
<5	37 757	1	1	1	1	1	1	1	1	
≥5	27 364	1.01 (.86–1.20)	0.91 (.56–1.48)	1.48 (.93–2.36)	0.65 (.38–1.12)	1.09 (.49–2.41)	1.24 (.59–2.63)	1.25 (.51–3.10)	0.98 (.51–1.87)	
AIDS at ART initiation										
No	50 919	1	1	1	1	1	1	1	1	
Yes	14 202	4.31 (3.55–5.23)	0.68 (.37–1.24)	2.84 (1.74–4.63)	1.90 (1.06–3.41)	1.18 (.47–2.98)	1.22 (.53–2.81)	0.51 (.15–1.73)	0.89 (.35–2.27)	
Period of HAART initiation										
1996–1999	19 720	1	1	1	1	1	1	1	1	
2000–2003	22 528	1.00 (.82–1.20)	0.77 (.43–1.41)	1.72 (.93–3.20)	0.91 (.48–1.72)	1.18 (.46–3.02)	1.02 (.44–2.40)	0.95 (.31–2.87)	0.26 (.10–.63)	
2004 onward	22 873	0.83 (.67–1.03)	0.98 (.55–1.75)	1.88 (1.00–3.53)	1.08 (.56–2.11)	0.58 (.19–1.75)	0.88 (.35–2.22)	1.40 (.49–4.04)	0.43 (.19–.99)	

Data are presented as hazard ratios (95% confidence intervals).

Abbreviations: ART, antiretroviral therapy; CVD, cardiovascular disease; HAART, highly active antiretroviral therapy; IDU, injection drug user; MSM, men who have sex with men.

^a^ Adjusted for transmission risk, sex, age, Viral Load, CD4, AIDS, year of ART initiation, stratified by cohort.

^b^ Only 10 accident/violent deaths in the first year of ART, therefore analysis not presented.

Table 4 shows associations of risk factors with cause-specific mortality after the first year of ART. Of 56 756 patients alive and in follow-up 1 year after starting ART, we excluded 9025 (15.9%) patients without 12-month measures of CD4 and viral load (n = 47 731). Of 2326 deaths after the first year, 373 could not be classified and 223 were from other causes. CD4 at 12 months was strongly inversely associated with subsequent rates of AIDS mortality, and also inversely associated with non-AIDS malignancy, non-AIDS infection, and liver-related death. Baseline CD4 was also strongly inversely associated with AIDS, non-AIDS infection, and liver-related mortality after the first year of ART, although the association with non-AIDS malignancy was attenuated compared with the first year. Lack of viral suppression at 12 months was associated with rates of AIDS (HR, 3.6 [95% CI, 3.0–4.3]), non-AIDS infection (HR, 3.3 [95% CI, 2.4–4.5]), heart/vascular (HR, 1.9 [95% CI, 1.2–3.1]), suicide (HR, 2.0 [95% CI, 1.2–3.4]), and liver-related death (HR, 1.5 [95% CI, 1.1–2.0]). An AIDS diagnosis prior to 12 months was associated with subsequent rates of AIDS (HR, 2.3 [95% CI, 1.9–2.7]) and non-AIDS infection (HR, 1.5 [95% CI, 1.1–2.0]) death.

**Table 4.** Adjusted^a^ Hazard Ratios From Cox Regression Models After the First Year of Antiretroviral Therapy (n = 47 731)

Risk Factor	No.	AIDS	Non-AIDS Malignancy	Non-AIDS Infection	Liver Related	CVD	Heart/Vascular	Suicide	Substance Abuse	Accident/ Other Violent
No. (%) of deaths		643 (28)	326 (14)	178 (8)	184 (8)	106 (5)	78 (3)	64 (3)	111 (5)	40 (2)
Transmission risk group										
MSM	16 227	1	1	1	1	1	1	1	1	1
IDU	7003	1.53 (1.22–1.91)	1.71 (1.19–2.45)	4.76 (2.94–7.71)	9.05 (5.75–14.24)	1.88 (1.03–3.40)	4.54 (2.32–8.86)	1.34 (.68–2.64)	5.87 (3.14–10.97)	2.68 (1.19–6.04)
Heterosexual	19 737	0.76 (.60–.96)	1.34 (1.01–1.77)	2.16 (1.33–3.51)	0.81 (.45–1.46)	0.82 (.47–1.41)	1.30 (.66–2.56)	0.68 (.33–1.41)	1.47 (.70–3.08)	0.35 (.11–1.07)
Other	4764	1.27 (.98–1.64)	1.14 (.76–1.72)	3.02 (1.74–5.25)	2.22 (1.20–4.10)	1.81 (1.01–3.24)	2.11 (.96–4.65)	0.46 (.15–1.40)	2.45 (1.14–5.24)	1.71 (.64–4.54)
Sex										
Male	34 468	1	1	1	1	1	1	1	1	1
Female	13 263	1.08 (.88–1.32)	0.56 (.40–.79)	0.70 (.47–1.04)	0.84 (.56–1.25)	0.89 (.51–1.56)	0.85 (.47–1.54)	0.67 (.31–1.46)	0.86 (.52–1.42)	1.01 (.41–2.49)
Current age (per decade)	47 731	1.18 (1.09–1.28)	2.08 (1.90–2.29)	1.46 (1.26–1.70)	1.55 (1.31–1.83)	2.12 (1.80–2.51)	1.87 (1.51–2.31)	1.12 (.86–1.46)	1.16 (.93–1.45)	1.23 (.88–1.71)
CD4 count at 12 mo, cells/µL										
<200	9020	1	1	1	1	1	1	1	1	1
200–349	12 794	0.38 (.31–.46)	0.76 (.57–1.01)	0.62 (.43–.90)	0.54 (.37–.79)	0.95 (.55–1.65)	0.92 (.51–1.65)	1.93 (.84–4.39)	0.47 (.26–.83)	1.25 (.50–3.10)
350–499	11 526	0.18 (.14–.24)	0.63 (.45–.87)	0.47 (.30–.74)	0.44 (.28–.69)	0.76 (.41–1.43)	0.59 (.29–1.19)	1.20 (.48–3.00)	0.78 (.46–1.34)	1.33 (.51–3.44)
500–749	10 028	0.15 (.10–.21)	0.59 (.41–.85)	0.34 (.19–.59)	0.36 (.22–.60)	1.08 (.58–2.00)	0.72 (.35–1.45)	1.32 (.53–3.29)	1.04 (.61–1.76)	0.66 (.21–2.13)
≥750	4363	0.18 (.11–.29)	0.59 (.36–.95)	0.59 (.31–1.12)	0.35 (.18–.70)	1.34 (.64–2.79)	0.45 (.15–1.34)	2.07 (.78–5.50)	0.45 (.18–1.10)	1.10 (.31–3.91)
Viral suppression at 12 mo										
Yes	34 695	1	1	1	1	1	1	1	1	1
No	13 036	3.61 (3.03–4.30)	1.07 (.83–1.38)	3.26 (2.37–4.50)	1.46 (1.07–1.99)	0.94 (.59–1.52)	1.93 (1.19–3.14)	2.01 (1.18–3.42)	1.36 (.91–2.03)	1.31 (.67–2.57)
AIDS at 12 mo										
No	36 111	1	1	1	1	1	1	1	1	1
Yes	11 620	2.28 (1.93–2.69)	1.26 (.99–1.61)	1.47 (1.06–2.04)	1.25 (.90–1.73)	1.12 (.71–1.76)	0.78 (.45–1.35)	0.44 (.20–.96)	1.05 (.66–1.68)	1.04 (.48–2.22)
Period of HAART initiation										
1996–1999	14 805	1	1	1	1	1	1	1	1	1
2000–2003	17 327	0.88 (.74–1.06)	0.74 (.57–.97)	1.44 (1.02–2.02)	0.67 (.47–.95)	0.86 (.54–1.39)	0.75 (.44–1.28)	0.77 (.43–1.40)	0.86 (.56–1.32)	0.57 (.27–1.21)
2004 onward	15 599	1.04 (.78–1.37)	1.04 (.71–1.50)	0.91 (.47–1.75)	0.56 (.30–1.07)	1.61 (.82–3.16)	0.79 (.34–1.81)	0.85 (.33–2.19)	0.58 (.27–1.23)	0.64 (.17–2.38)
CD4 count at ART initiation^b^, cells/µL										
<50	7622	1	1	1	1	1	1	1	1	1
50–99	4786	0.73 (.58–.93)	1.18 (.82–1.70)	0.74 (.44–1.23)	1.04 (.63–1.72)	0.73 (.33–1.62)	0.68 (.29–1.60)	0.77 (.22–2.67)	1.54 (.74–3.21)	1.61 (.46–5.63)
100–199	9646	0.60 (.48–.75)	0.91 (.64–1.31)	0.78 (.50–1.21)	0.82 (.51–1.31)	1.19 (.63–2.25)	0.81 (.40–1.65)	0.72 (.25–2.07)	1.28 (.64–2.56)	1.33 (.41–4.31)
200–349	14 551	0.39 (.30–.50)	0.75 (.52–1.08)	0.53 (.33–.87)	0.77 (.48–1.24)	1.12 (.59–2.14)	0.50 (.24–1.08)	1.11 (.44–2.84)	1.35 (.68–2.66)	1.17 (.36–3.84)
350–499	6470	0.24 (.17–.34)	0.67 (.42–1.06)	0.46 (.25–.85)	0.80 (.46–1.38)	0.79 (.34–1.82)	0.47 (.19–1.18)	1.44 (.53–3.89)	1.47 (.68–3.17)	1.14 (.30–4.26)
≥500	4656	0.19 (.12–.30)	0.98 (.61–1.56)	0.59 (.31–1.12)	0.38 (.18–.82)	1.47 (.67–3.25)	0.90 (.38–2.13)	2.10 (.77–5.68)	2.04 (.94–4.43)	1.52 (.40–5.80)

Data are presented as hazard ratios (95% confidence intervals).

Abbreviations: ART, antiretroviral therapy; CVD, cardiovascular disease; HAART, highly active antiretroviral therapy; IDU, injection drug user; MSM, men who have sex with men.

^a^ Adjusted for transmission risk, sex, age, 12-month Viral Load, CD4, and AIDS status, and year of ART initiation, stratified by cohort.

^b^ This is a separate model adjusted for baseline CD4, viral load, and AIDS status, plus all other variables listed in table.

The association of presumed transmission via injection drug use (compared with MSM) with AIDS death was stronger in subsequent years of ART (HR, 1.5 [95% CI, 1.2–1.9]) than the first year (HR, 1.1 [95% CI, 0.9–1.5]). IDUs also experience higher rates of death from non-AIDS malignancy (HR, 1.7 [95% CI, 1.2–2.5]), non-AIDS infection (HR, 4.8 [95% CI, 2.9–7.7]), and CVD (HR, 1.9 [95% CI, 1.0–3.4]) after the first year of ART; the strong association of injection drug use with liver-related death was lower after (HR, 9.1 [95% CI, 5.8–14.2]) than during the first year of ART. Compared with MSM, heterosexual transmission was associated with higher rates of non-AIDS malignancy (HR, 1.3 [95% CI, 1.0–1.8]), and infection-related (HR, 2.2 [95% CI, 1.3–3.5]) death. Sex differences in cause-specific mortality rates were less pronounced after than during the first year of ART. Age was most strongly associated with death from CVD and non-AIDS malignancy. The effect of age on AIDS deaths declined over time, suggesting that older people are more vulnerable to AIDS in the first year of ART. There was little evidence that rates of cause-specific mortality changed over calendar time, having adjusted for other risk factors. CVD, heart/vascular, and substance abuse deaths were not strongly related to conventional HIV risk factors. Sensitivity analyses accounting for competing risks showed similar associations between risk factors and specific causes of mortality both during and after the first year of ART (data not shown).

## DISCUSSION

Based on data from a large, multinational cohort collaboration in which causes of death were classified using standardized procedures, both the distribution of causes of death and their associations with patient characteristics differed substantially with time since starting ART. Whereas rates of AIDS death decreased substantially with time since starting ART, rates of non-AIDS malignancy death increased. Higher mortality in men than women during the first year of ART was mostly driven by higher rates of non-AIDS malignancy and liver-related death: between-sex differences decreased with increasing time on ART. Associations with age were strongest for death from CVD, heart/vascular causes, and malignancy. In these data, 46% of patients started ART with a CD4 count <200 cells/µL. There was a persistent role of CD4 count at baseline and 12 months after starting ART in predicting rates of death from AIDS, non-AIDS infection, and non-AIDS malignancy. Lack of viral suppression on ART was strongly associated with death from AIDS and non-AIDS infection, and was also associated with a number of other causes of death. Patients with presumed transmission via injection drug use had persistently high rates of liver-related death, and persistently higher rates of both AIDS and other non-AIDS causes of death.

There were several strengths and limitations to this study. Patients were from diverse populations in clinical care across Europe and North America [18]. We compiled a dataset with follow-up on >65 000 patients who experienced >4000 deaths. Thanks to a team of experienced HIV clinicians using standardized procedures, 3574 (84%) deaths were classified as due to specific causes [19]. Disagreements were carefully resolved via panel discussion. Coding was done retrospectively and without complete patient histories, so there will be some misclassification. Classification of cause of death based on autopsy remains the gold standard: clinical classifications may not correlate well with classifications from autopsy reports [20, 21]. However rates of autopsy are diminishing over time [8]. Not all deaths could be classified, which will bias estimates of cause-specific mortality rates downward. Application of a stepwise algorithm to code deaths as AIDS or non-AIDS related could allow broad coding of deaths where specific cause is missing [22]. Data on established risk factors for chronic diseases were not available for all patients and therefore we were unable to estimate their contributions to cause-specific mortality. For example, we do not know whether patients smoke tobacco or abuse alcohol. Some patients classified in the “other” transmission group are likely to have had transmission via injection drug use. Data on ART adherence was not available: this information might provide further insights into patterns of cause-specific mortality but is captured to an extent by viral suppression at 12 months.

The EuroSIDA cohort [23] found little evidence for increased rates of all-cause or non-AIDS-related deaths with long-term cumulative combination ART exposure. By contrast, we found evidence that deaths from non-AIDS malignancy increased with time since ART. An Italian study showed that malignancies, infections, and end-stage liver disease were leading causes of death among PLWH [4]. Studies of the HIV-infected population in France and Switzerland have shown the increasing importance of non-AIDS malignancies, liver disease, and CVD over calendar time [8, 15, 24] and this was seen for non-AIDS malignancies in our previous work [9]. Prevalence of tobacco smoking is known to be higher among PLWH and may contribute substantially to rates of death from CVD and some cancers [25, 26]. Other established chronic disease risk factors may also be more prevalent in HIV-infected populations.

As patients become increasingly treatment experienced, monitoring patterns of causes of death over longer durations of ART will be important for optimizing management of PLWH, many of whom develop comorbidities [27]. Understanding changes in cause-specific deaths with ART duration can help direct appropriate screening procedures, risk assessment, and preventive treatment for PLWH. The well-known high early mortality from AIDS [28, 29] is best addressed by more timely HIV diagnosis and earlier treatment [30]. The persistent role of lower CD4 counts and lack of viral suppression in predicting both AIDS and non-AIDS death after the first year of ART emphasizes the importance of maintaining adherence to therapy and ensuring patients are switched from failing regimens. The association of injection drug use with AIDS deaths was stronger after the first year, which may suggest a role of nonadherence to ART. Mortality later in the course of treatment is increasingly due to non-AIDS malignancies, liver disease, and CVD, whose occurrence also increases with age in HIV-uninfected individuals. Continuing study of cause-specific mortality is required to clarify whether such mortality arises from effects of ART, prolonged exposure to the virus, consequences of restoration of CD4 counts after severe immunosuppression, or whether such mortality mainly reflects aging and non-HIV risk factors. Large studies would be required to compare age-standardized mortality rates and risk factors for HIV-infected and -uninfected populations, to quantify excess cause-specific mortality among HIV-infected individuals once known risk factors are accounted for [27].

To better differentiate mortality risks for those on ART, it will be important to monitor “non-HIV” biomarkers as well as CD4 and viral load. The VACS Index, which includes markers for anemia, liver injury, renal insufficiency, and chronic viral hepatitis as well as factors prognostic for HIV progression, can identify patients at high risk of mortality [31, 32]. Better treatment of hepatitis B and C coinfection [33, 34], interventions to decrease harm from alcohol or illicit drug abuse, and smoking cessation programs [35] potentially offer opportunities to decrease the risk of diseases that are prevalent among PLWH. US treatment guidelines recommend screening for hepatitis C before starting ART for all PLWH, but annual screening is only recommended for certain subgroups [36]. The European AIDS Clinical Society guidelines recommends screening for certain non-AIDS malignancies in subgroups of PLWH, annual screening for hepatitis C, and management of CVD risk as part of routine clinical care [37]. However, guidelines are not always followed, and improved management of chronic diseases will require close collaboration between specialists.

## Supplementary Data

Supplementary materials are available at *Clinical Infectious Diseases* online (http://cid.oxfordjournals.org). Supplementary materials consist of data provided by the author that are published to benefit the reader. The posted materials are not copyedited. The contents of all supplementary data are the sole responsibility of the authors. Questions or messages regarding errors should be addressed to the author.

Supplementary Data

## APPENDIX

***List of contributing cohorts.*** Sixteen cohorts contributed data on causes of death: the French Hospital Database on HIV (FHDH) and the Aquitaine Cohort (France), the AIDS Therapy Evaluation project Netherlands (ATHENA), Italian Cohort of Antiretroviral-Naïve Patients (ICONA), the Swiss HIV Cohort Study (SHCS), the Köln/Bonn Cohort (Germany), the EuroSIDA study (33 countries in Europe and Argentina), the HAART Observational Medical Evaluation and Research (HOMER) and the Southern Alberta Clinic Cohort (Canada), the Proyecto para la Informatizacion del Seguimiento Clinico-epidemiologico de la Infeccion por HIV y SIDA (PISCIS), Cohorte de la Red de Investigación en Sida (CoRIS) cohort and the VACH cohort (Spain), the 1917 clinic cohort University of Alabama, the University of Washington HIV cohort, the HIV Atlanta Veterans Affairs Cohort Study (HAVACS), and the Vanderbilt-Meharry Center for AIDS Research, Nashville, Tennessee.
