# Supplementary material for: Impact of Risk Factors for Specific Causes of Death in the First and Subsequent Years of Antiretroviral Therapy Among HIV-Infected Patients
Source: Clin Infect Dis. 2014 Apr 24;59(2):287–97. doi: 10.1093/cid/ciu261 (PMC4073781; doi:10.1093/cid/ciu261)
Supplement: Supplementary Data [file supp_59_2_287__index.html]

Impact of risk factors for specific causes death in the first and subsequent years of ART among HIV-infected patients — Impact of Risk Factors for Specific Causes of Death in the First and Subsequent Years of Antiretroviral Therapy Among HIV-Infected Patients — Impact of Risk Factors for Specific Causes of Death in the First and Subsequent Years of Antiretroviral Therapy Among HIV-Infected Patients — Supplementary Data 

# Impact of Risk Factors for Specific Causes of Death in the First and Subsequent Years of Antiretroviral Therapy Among HIV-Infected Patients

## Supplementary Data

Supplementary Data

**Files in this Data Supplement:**

- Supplementary Data - Docx file
