# Supplementary material for: Impact of Risk Factors for Specific Causes of Death in the First and Subsequent Years of Antiretroviral Therapy Among HIV-Infected Patients
Source: Clin Infect Dis. 2014 Apr 24;59(2):287–97. doi: 10.1093/cid/ciu261 (PMC4073781; doi:10.1093/cid/ciu261)
Supplement: Supplementary Data [file supp_ciu261_ciu261supp.docx]

Coding causes of death

To further ensure the consistency of coding between clinicians, rules were additionally developed to aid the assignment of CoDe categories. Death due to Hodgkin lymphoma was classified as AIDS related. If a patient was positive for hepatitis C or B antibodies at baseline and died of “liver cancer,” we assumed the cancer was secondary to hepatitis. Deaths described as accidental/violent with a history of self-harm in ICD-9 were coded as suicide (CoDe 17). If it was unclear whether death was attributable to suicide, the less specific code of accidental/violent death (CoDe 16) was assigned. Deaths described as due to cardiac arrest or respiratory failure with no other information were coded as unknown (CoDe 92). Deaths with insufficient information to achieve a consensus were coded as cause unknown (CoDe 92). Further details for the rules on coding are given below.

**ART-CC rules for coding causes of death**

Choice of deaths for adjudication

When there is broad agreement between the clinician’s code and the code from the computer program, but the clinician’s code is more precise, we will use the clinician coding without further adjudication.

For hepatitis codes, if the computer program has generated a more specific code than the clinician’s (i.e. to indicate hep C or hep B) then we will use the code from the programme without further adjudication.

AIDS, AIDS infection, AIDS malignancy and other infections

1. “Low” CD4 count, which influences whether we code deaths as AIDS, is classified as <100 cells/mm^3^.
2. CoDe 01.1 (“AIDS: Infection”) should be used if there was an AIDS-defining infection within the year prior to death, (regardless of CD4) if “HIV infection” is mentioned as most responsible or underlying cause of death.
3. Even if there was no AIDS-defining infection or AIDS-defining malignancy in the last year, if there is a CD4<100 and HIV is given as **both** cause of death and underlying cause it will be coded as 01 (“AIDS”).
4. If the patient was off therapy when last seen, and the CD4 count was low (< 100), then the death may be coded as 01 (“AIDS”) even if the last available information was between 12 and 18 months ago.
5. If the cause of death is poorly defined, but there was an AIDS event within 2 months of death, this can be coded 01: AIDS.
6. If there is an ICD code of B218 (“HIV disease resulting in malignant neoplasm”), deaths should be coded as 01.2 (“AIDS: Malignancy”)
7. ICD codes of C82 to C85 (non-Hodgkin’s lymphoma) should be coded as 01.2 (“AIDS: malignancy”)
8. Deaths due to Invasive Cervical Cancer (ICC) or Kaposi’s Sarcoma should be coded 01.2: AIDS malignancy.
9. When there is an ill-defined cause of death and CD4 > 100, but the patient was previously diagnosed with NHL this should be this should be coded 01.2: AIDS Malignancy.
10. If the ICD10 code gives an ill-defined cause of death (i.e. code R99) and the patient had TB, this should be coded as 01: AIDS, regardless of CD4 count. Additionally, if the TB was in the last year, this should be coded as 01.1: AIDS Infection.
11. Wasting disease should be classified as AIDS 01 (not AIDS infection 01.1).
12. Deaths due to Endocarditis and Myocarditis should be coded as 02 (“Infection”) and sub classified as 02.1 if known to be bacterial.
13. Mycobacteria should be coded 01 “AIDS” in the absence of other reasons for death.
14. There is no need to use CoDe 02.2 (“Infection: others”). Just use CoDe 02 (“Infection”)
15. CoDe 02.1 (“Infection: bacterial”) should only be used if there is mention of a bacterial disease or of “sepsis” which is a term used almost exclusively for bacterial infections. Otherwise just use CoDe 02 (“Infection”). If cause of death is sepsis/septicaemia, code as 02.1 even if recent AIDS and/or low CD4

Rules for patients with an ICD-10 B207 or B208 code (“HIV-disease resulting in multiple infections” and “HIV-disease resulting in other infectious and parasitic diseases”)

1. If CD4 is low ( <100 )and the patient had pneumonia (unspecified), but there is no mention of sepsis, this should be coded as 01 (“AIDS”)
2. If CD4 is unknown or ‘high’ and the patient had pneumonia (unspecified), but there is no mention of sepsis, this should be coded as 02 (“Infection”).
3. If CD4 is low (<100) or unknown, and there is no mention of any cause of death other than HIV (B208 or B24 (“Unspecified HIV disease”)), this should be coded as 01 (“AIDS”)
4. For patients with a disagreement between CoDe 01 and 02, if CD4 is low (<100) and patient has 3 causes of death listed as B208, B24 and B218 (“HIV disease resulting in other malignant neoplasms”) this should be coded 01 (“AIDS”)

Liver-related deaths

1. If there is no evidence of Hep C (either Hep C positive or IDU as stated in paper), or Hep B, deaths from liver failure should be coded as 14 Liver Failure. If there is evidence of Hep C (or IDU), such deaths should be coded as 03.1 (“Chronic viral hepatitis: HCV”). If there is evidence of Hep B, such deaths should be coded as 03.2 (“Chronic viral hepatitis: HBV”).
2. If a liver-related death also includes alcohol in the cause of death, this should be coded 14.
3. We will not attempt to differentiate between liver failure and cirrhosis subcodes (3.1.1 and 3.1.2, and 3.2.1 and 3.2.2). We will code only as 3.1 or 3.2

General rules

1. If codes suggest that death was due to a post-operative infection, this should be coded as 90 “Other causes” with an additional note.
2. Intracerebral haemorrhage should be coded as 09 (stroke) rather than 23 (CNS disease)
3. For overdose deaths, patients with Hep C or IDU should be coded as 19.2 (“Substance abuse: intravenous drug use”). Patient who are not Hep C positive and not IDU should be coded as 19 (“Substance abuse (active))”
4. Deaths with ICD-10 code X44 (“Substance use: active intoxication”) should be coded as 19.2 (“Substance abuse: chronic intravenous drug use) use if the patient is hep C positive or IDU
5. If only one ICD 9/10 code exists for a patient, and this suggests that death was due to alcohol, this should be coded as 19.1 ("Chronic Alcohol Abuse")
